# Recent developments in biofeedback for neuromotor rehabilitation

**DOI:** 10.1186/1743-0003-3-11

**Published:** 2006-06-21

**Authors:** He Huang, Steven L Wolf, Jiping He

**Affiliations:** 1 Center for Neural Interface Design in The Biodesign Institute, and Harrington Department of Bioengineering, Arizona State University, Tempe, Arizona, 85287, USA; 2 Department of Rehabilitation Medicine, Emory University School of Medicine, Atlanta, Georgia, 30322, USA; 3 Huazhong University of Science and Technology, Wuhan, China

## Abstract

The original use of biofeedback to train single muscle activity in static positions or movement unrelated to function did not correlate well to motor function improvements in patients with central nervous system injuries. The concept of task-oriented repetitive training suggests that biofeedback therapy should be delivered during functionally related dynamic movement to optimize motor function improvement. Current, advanced technologies facilitate the design of novel biofeedback systems that possess diverse parameters, advanced cue display, and sophisticated control systems for use in task-oriented biofeedback. In light of these advancements, this article: (1) reviews early biofeedback studies and their conclusions; (2) presents recent developments in biofeedback technologies and their applications to task-oriented biofeedback interventions; and (3) discusses considerations regarding the therapeutic system design and the clinical application of task-oriented biofeedback therapy. This review should provide a framework to further broaden the application of task-oriented biofeedback therapy in neuromotor rehabilitation.

## Review of early biofeedback therapy

Biofeedback can be defined as the use of instrumentation to make covert physiological processes more overt; it also includes electronic options for shaping appropriate responses [1-3]. The use of biofeedback provides patients with sensorimotor impairments with opportunities to regain the ability to better assess different physiological responses and possibly to learn self-control of those responses [4]. This approach satisfies the requirement for a therapeutic environment to "heighten sensory cues that inform the actor about the consequences of actions (forward modeling) and allows adaptive strategies to be sought (inverse modeling)" [5]. The clinical application of biofeedback to improve a patient's motor control begins by re-educating that control by providing visual or audio feedback of electromyogram (EMG), positional or force parameters in real time [6,7]. Studies on EMG biofeedback indicated that patients who suffer from sensorimotor deficits can volitionally control single muscle activation and become more cognizant of their own EMG signal [8,9]. The neurological mechanisms underlying the effectiveness of biofeedback training are unclear, however. Basmajian [10] has suggested two possibilities: either new pathways are developed, or an auxiliary feedback loop recruits existing cerebral and spinal pathways. Wolf [7], favoring the latter explanation, posited that visual and auditory feedback activate unused or underused synapses in executing motor commands. As such, continued training could establish new sensory engrams and help patients perform tasks without feedback [7]. Overall, biofeedback may enhance neural plasticity by engaging auxiliary sensory inputs, thus making it a plausible tool for neurorehabilitation.

From the 1960s to the 1990s, many studies investigated the effects of biofeedback therapy on the treatment of motor deficits in the upper extremity (UE) [11-18] and lower extremity (LE) [19-30] by comparing the effects of biofeedback training with no therapy or with conventional therapy (CT). Patients included those with strokes [12-14,18-24,26-31], traumatic brain injury [15,32], cerebral palsy [25,33,34], and incomplete spinal cord injury [16,17]. Because this review focuses on new technologies and to avoid repeating past study findings, we only summarize briefly the main characteristics of clinical applications of biofeedback for neuromotor therapy.

The applied physiological sources to be fed back included EMG [11-14,17,22-24,26,29,30,35], joint angle [20,29,31,36], position [37,38], and pressure or ground reaction force [39-41]. EMG was employed as a primary biofeedback source to down-train activity of a hyperactive muscle or up-train recruitment of a weak muscle, thus improving muscular control over a joint [6]. Angular or positional biofeedback was used to improve patients' ability to self-regulate the movement of a specific joint. Parameters such as center of gravity or center of pressure, derived from ground reaction forces measured by a force plate, were often used as feedback sources during balance retraining programs.

Although EMG was used most frequently, it may not always be the best biofeedback source for illustrating motor control during dynamic movement. For example, Mandel et al. [26] demonstrated that with hemiparetic patients, rhythmic ankle angular biofeedback therapy generated a faster walking speed than EMG biofeedback without increasing the patients' energy cost.

Regardless of the type of biofeedback employed cues in past designs were usually displayed in a relatively simplistic format with analog, digital or binary values. The feedback is indicated through visual display, auditory pitch or volume, or mechanical tactile stimulation, with the last arising from a simple mechanical vibrating stimulator attached to the skin [33].

In addition, patients in older biofeedback studies learned to regulate a specific parameter through a quantified cue while in a static position, or they performed a simple movement unrelated to the activities of daily living (ADL) [13,23,24,30]. We define this as "static biofeedback"; EMG is a classic form. Traditional EMG biofeedback studies showed that patients can improve voluntary control of the activity of the trained muscle and/or increase the range of motion of a joint that the trained muscle controls [12,22,23]. The overall effect of this type of biofeedback training on motor recovery is inconsistent, however. Meta-analyses of studies on stroke patients exemplify this [3,42-44]. Schleenbaker and Mainous [42] showed a statistically significant effect from EMG biofeedback, whereas the other studies concluded that little, if any, improvement could be definitively determined [3,43,44]. As is true for many meta-analyses, contradictory conclusions might result from different assessment criteria or from incongruities in the specification of performance measurements. Schleenbaker and Mainous [42] included non-randomized control studies in their analysis; other analyses considered data only from randomized controlled trials (RCT) [3,43,44].

Diversity among outcome measurements also promotes alternative conclusions among biofeedback studies. Glanz *et al*. [44] used range of motion as an assessment criterion, while the other analyses used functional scores. EMG biofeedback yielded positive effects if the outcome measurement was related to control of a specific muscle or joint [12,22,23,45]. Most results and reviews of static biofeedback therapy, however, do not demonstrate that it leads to significant motor function recovery [16,18,30,43,46]. For example, Wolf *et al *. down-trained the antagonist and up-trained the agonist of an elbow extensor by static EMG biofeedback. This did not help stroke patients to extend their elbows during a goal-directed reaching task, and muscle co-contraction still occurred during coordinated movement [18]. Furthermore, the application of static EMG biofeedback training to LE of hemiplegic patients did not affect functional walking [30,43]. Static EMG biofeedback therapy may thus produce only specific and limited effects on motor function recovery [47].

Variables such as the site or size of the brain lesion, the patient's motivation during therapy, and his/her cognitive ability may influence the effectiveness of biofeedback or any therapy. Moreland and colleagues [3,43] included in their meta-analyses studies with control groups that received conventional physical therapy, whereas the other two reports analyzed studies with no therapy in the control group. The latter are potentially biased in favor of biofeedback therapy. These inconsistent experimental protocols surely contributed to the contradictory conclusions [7]. A better design for experimental protocols to evaluate the efficacy of biofeedback therapy needs to be adopted [7,43,44]. Randomized controlled trials (RCT) are the gold standard for obtaining a statistically acceptable conclusion; double blind experimental designs best eliminate bias [7]. Given contemporary ethical considerations, however, double blind feedback studies in which neither the patient nor the evaluator knows if the feedback was bogus or real are probably impractical.

Biofeedback provided during function-related task training is defined as task-oriented or "dynamic biofeedback" (in comparison to static biofeedback). While several past studies employed a form of dynamic biofeedback for rehabilitation of postural control or walking [26,29,37] or with reaching and grasping tasks [48], the applied technology and training protocol were relatively simplistic by today's standards.

## Current developments in biofeedback in neurorehabilitation

### New concept: from static to task-oriented biofeedback

One major goal of rehabilitation is for patients with motor deficits to reacquire the ability to perform functional tasks. This is intended to facilitate independent living. Contemporary opinion on motor control principles suggests that improvement in functional activities would benefit from task-oriented biofeedback therapy [5,30,43,46]. Because any functional ADL task explicitly requires an interaction between the neuromuscular system and the environment, effective motor training should incorporate movement components and an environment that resemble the targeted task in the relevant functional context [49,50]. Thus, task learning must be linked to a clearly defined functional goal. In neuromotor rehabilitation, task-oriented training encourages a patient to explore the environment and to solve specific movement problems [5]. Therefore, effective biofeedback therapy for patients with motor deficits should re-educate the motor control system during dynamic movements that are functionally-goal oriented rather than relying primarily upon static control of a single muscle or joint activity.

Several studies have focused on repetitive task-oriented training in which real-time biofeedback is provided during task performance [20,29,35,37,38,43,51,52]. However, a task-oriented feedback therapy approach requires overcoming several difficulties.

During the training of functional tasks, it is important to choose the best information or variable to feed back. Muscle activity is not always superior [26]. The choice of a biofeedback vehicle should depend upon the motor control mechanism, training task, and therapeutic goal [46]. Assume that the training task for a hemiparetic patient is to reach for and grasp a cup of coffee using only the affected arm. Recent motor control models suggest that the brain may control limb kinematics in a reaching task by shifting the equilibrium points [53] or creating a "virtual trajectory" of the end-point [54], instead of scaling individual muscle activity patterns [55]. Therefore, hand trajectory may be a more viable feedback variable than muscle activity for reaching related tasks [56]. In addition to hand transportation, successful reaching and grasping actions also require a hand orientation permitting the alignment of the finger-thumb opposition axis with that of the object [57-59], and control of the finger grip aperture [60]. These variables should be considered when designing dynamic feedback options to facilitate limb control [61].

Using multiple indices brings out another difficulty, however: how does the system feed back multiple sources of information to patients whose cognition and perception may also be impaired without overloading them with information? If the variables were displayed with traditional abstract and quantitative cues, either visual or auditory, patients may not pay attention to all of them. Inevitably, the ability to process multiple sources will become overburdened [50]. The patient may become confused and distracted, resulting in rapid deterioration of task performance. Designing a biofeedback system that overcomes the "information overloading" obstacle for task retraining is both a technical and conceptual challenge.

Therefore, an effective task-oriented biofeedback system requires orchestrated feedback of multiple variables that characterize the task performance without overwhelming a patient's perception and cognitive ability. A usable system of biofeedback for repetitive task training in neuromotor rehabilitation requires sophisticated technology for sensory fusion and presentation to be available for adoption. Fortunately, technology in this area has advanced considerably since early studies on biofeedback.

### New technologies and applications for task-oriented biofeedback training

#### Information fusion

An information/sensory fusion approach is one way to reduce information overload to patients during biofeedback therapy. Information fusion involves integrating a dynamic and volatile flow of information from multimodal sources and multiple locations to determine the state of the monitored system. [62-64]. Information fusion can occur at different conceptual levels, including data acquisition (numerical/symbolic information), processing (such as features and decisions), and modeling [62]. This approach is beneficial because it mimics human intelligence. As a result, it improves the robustness of machine perception or decision making to monitor or control dynamic systems or those with uncertain states [62].

Information fusion is analogous to augmented feedback information given by therapists while training patients to perform a task. It can be designed to identify the patient's performance based on sensing data and to decide the usefulness of providing feedback through cues. The composite variables that information fusion constructs from multiple information flows provide intuitive and easily presented information relevant for knowledge of performance (KP) and therapeutic result.

**Figure 1:**
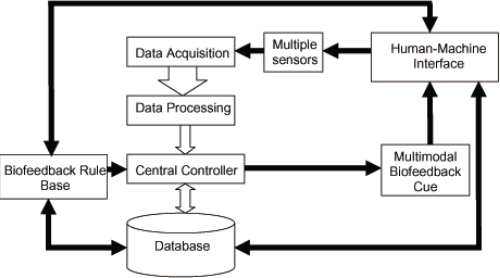
**General architecture of a multisensing task-oriented biofeedback system**. The detailed functions of each module in the flowchart are described in Table 1.

Figure 1 summarizes the general architecture of a task-oriented biofeedback system with multimodal sensor inputs [65,66]. Table 1 lists the function of each module shown in Figure 1. The central controller is the system kernel and contains the fusion algorithms. It receives processed data observed or derived from sensors, a priori knowledge from a database or data storage, and biofeedback rules from the rule base. The embedded fusion algorithm recognizes the current state of the performance based on these inputs and makes decisions for the feedback display.

**Table 1:** Function of Basic Modules in Multisensing Biofeedback Systems for Task Training.

*Component*	*Function*
Multiple Sensors	Multiple sensors transform various physiological or movement related information into recordable electronic signals.
Data Acquisition	Analog signals from multiple sensors are sampled, quantified and streamed into a control system.
Data Processing	The digital filter smoothes the data. The embedded algorithm or mathematical model can derive the secondary parameters as biofeedback indices.
Central Controller	The central controller is the kernel of the system. This module receives data from multiple sensors. Based on the biofeedback rules and user's pervious performance, the fusion algorithm in the controller identifies the participant's current state of task performance and decides the cue display.
Biofeedback Rule Base	This module stores a set of rules or criteria that can be defined by therapist via user interface or by prior knowledge of performance contained in the database. The rules or criteria are elements of the fusion algorithm. Decision making regarding the feedback display must obey these rules.
Multimodal Biofeedback Cue	This component configures the display hardware such as the screen, speaker, and haptic device. The program controls the display of augmented multimodal feedbacks based on commands from the controller.
Database	The database functions the same as traditional memory but with a more efficient structure for data management. It stores the parameters that are important to quantitatively evaluate the motor performance of patient. The controller and rule base access the database, query the patient's prior performance, and then adjust the feedback parameters and display. The database also allows direct access from authorized users.
Human-Machine Interface	This module configures the operation setting, rule choosing, etc. Through the human-machine interface, clients can customize the biofeedback training program based on their preferences. Authorized therapists or clients can access the record of a specific patient from the database to evaluate progress toward recovery.

The appropriate sensors to use in a biofeedback system depend on the training task and therapeutic goal. Ever-increasing processing power allows both data streams from multiple sensory sources and instant displays of the parameters derived by a complex algorithm or mathematical model. For instance, biomechanical models have been applied in several task-oriented biofeedback studies to calculate and feed back several variables in real time. These include joint angles and their derivatives from a motion capture camera [67,68], the configuration of fingers from an RMII Glove sensitive to fingertip positions [69], and the patient's self-generated joint torque from force and angle sensors [70].

A database is classically defined as a collection of information organized efficiently for data storage and query [71]. The biofeedback rule database contains rules that define how sensory information will be processed, how decisions will be made, and in what format information will be presented to the patient or therapist. They often take the form of direct mapping from sensory information to various types of augmented feedback, such as visual, auditory or tactile. Other rules are complex models that process the sensory information before feedback. These rules can be stored with raw data and should be updated and expanded as technology or knowledge advance. A simple device may only require data storage, while a complicated fusion algorithm may require the execution of data mining algorithms to obtain a patient's previous performance as prior knowledge, and then adjust the rule and decision criteria to form a user specific training protocol and interface [72].

Previous studies typically used a limited number of sensors so that the data fusion method and the structure of the applied biofeedback system were relatively simple [29,35,73]. For example, one study retrained spinal injured patients to correct a Trendelenburg gait [35]. The microcontroller-based portable biofeedback device integrated the data from EMG and insole pressure sensors, classified the patient's gait into "proper," "improper due to slow walking speed," or "improper due to low muscle activities during the swing phase," and then fed back the classification to patients through different auditory tones. In this case, the data fusion algorithm was equivalent to a classifier with manually set threshold. The biofeedback rules simply mapped a movement condition to a type of auditory tone. For a complicated task-oriented biofeedback system with more sensor inputs and intelligent monitoring and control, effective data fusion may require more sophisticated algorithms, such as artificial neural networks or a fuzzy logic based approach [74].

Two examples that apply complex multisensing systems and fusion algorithms are real-time movement tracking [75,76] and movement pattern recognition [67]. One reported multisensing system included magnetic, angular rate, and gravity sensors to track the 3-D angular motion of body segments. The sensory fusion employed a quaternion-based Kalman filter [75,77]. The movement status was fed back by animating a virtual human on the screen. In another study, a Kalman filter-based fusion algorithm fused data from a tri-axis accelerometer, gyro and magnetometer to more accurately track the position and orientation of human body segments [76]. The authors proposed that the system could be applied to virtual reality for medicine without discussing details.

In addition, a research team from the Arts, Media, and Engineering program at Arizona State University applied information fusion to an interactive art performance. They developed a fusion algorithm to recognize gesture patterns presented by dancers in real-time. The information was then fed back through digital graphics and sounds that reacted to, accompanied, and commented on the choreography [78]. A motion capture system with multiple cameras was used to monitor the position in 3D space of markers attached to a dancer. Postural features such as joint angles were extracted and then fused for recognition of movement patterns [67]. Due to variations in dancers' morphology and execution of the same gestures, a database was developed to store fusion algorithms in addition to customized parameters that allowed the algorithm to adapt to different users. However, none of these studies reported technical details on the implemented fusion algorithms [67,75,76].

Although information fusion is a potentially powerful tool for advanced biofeedback systems integrating multimodal and multisensor information, the challenge of determining the most appropriate and effective means to provide feedback remains.

### Virtual reality: technology and application

#### Multimedia based cue design for task-oriented biofeedback

A challenge in neuromotor rehabilitation is to identify the best methods to provide repetitive therapy for task training; these should involve multimodal processes to facilitate motor function recovery [61]. Task-oriented biofeedback therapy might be more widely effective if the biofeedback cues were: (1) multimodal, so perceptive and cognitive functions are involved in the physical therapy; (2) attractive and motivating, to keep the subject attentive; and (3) easy-to-understand, to avoid the information overloading problem. Multimedia based technology can be used to design biofeedback cues possessing these features. Multimedia uses computerized graphics/animation, sound, and/or haptic stimulation to immerse the user in a constructed virtual environment. This technology is thus called virtual reality (VR) in many studies.

**Figure 2:**
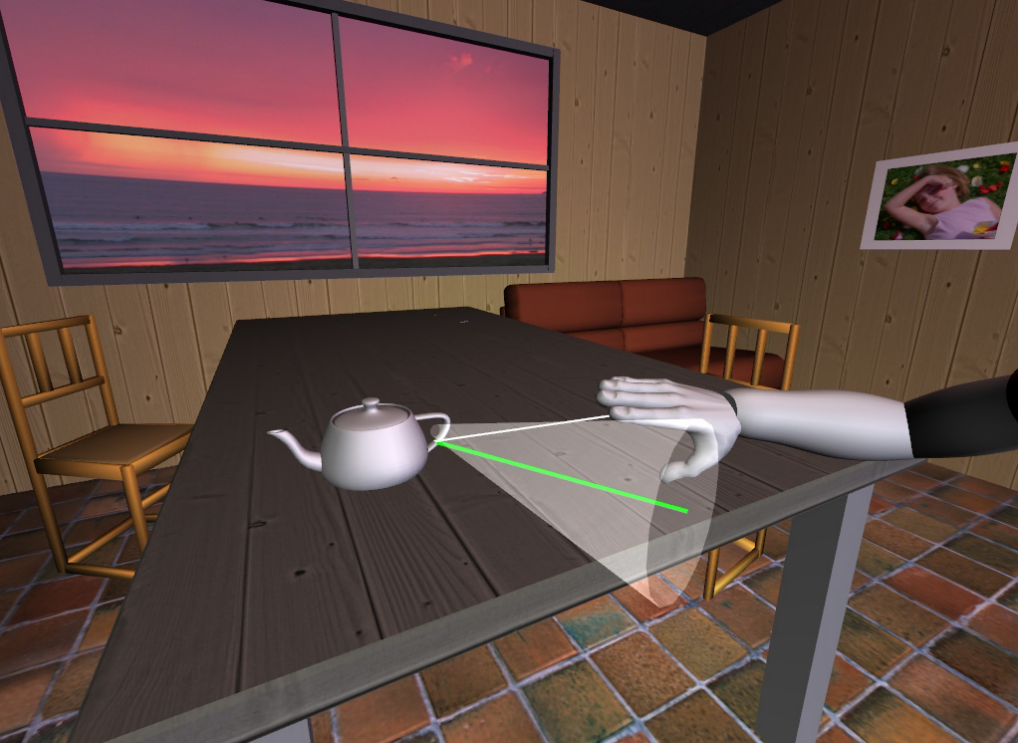
**Virtual environment design**. The design of a virtual living room is illustrated. The virtual arm animates the patient's arm movement in real time. The patient can explore the virtual environment and perform the goal-directed reaching task. The green line indicates the ideal trajectory. The cone shape constrains the spatial error of endpoint position and provides direct knowledge of performance [72].

Multimedia environments can offer real-life experiences by providing visual, auditory and physical interactions in an engaging manner. This may make them more effective than classic biofeedback presentation methods for task-oriented therapy. For example, a "room" scenario is designed to simulate ADL [72,73,79]. In the virtual room patients can practice functional tasks such as making coffee, pouring water into a glass [73], and reaching and grasping an object on a table or shelf (Figure 2) [79,80]. However, the real therapeutic benefits of these systems remain to be proven by well designed clinical trials.

An immersive multimedia environment is ideal for multimodal sensory feedback. Visual feedback is easily accomplished via computer graphics. A 3D stereo visual environment can be created with head-mounted displays (HMD) or 3D monitors [82,83]. These methods may not be suitable for neuromotor therapy among patients with brain injury, however, because motion sickness, dizziness and visual problems may occur [84]. A large screen with depth reference frames to aid 3D perception is an alternative choice.

Surround sound can provide an immersive environment in which to provide auditory biofeedback. Sound is a very effective feedback source for temporal information; visual information works better for spatial feedback [72]. Auditory feedback can take the form of pleasant and captive music pieces rather than the simplistic and often annoying tones or beeps in older biofeedback studies. Studies have shown that music can synchronize motor outputs [85,86], improve the motor coordination of Parkinson patients [87], and enhance motor learning in a patient with large-fiber sensory neuropathy [86].

**Figure 3:**
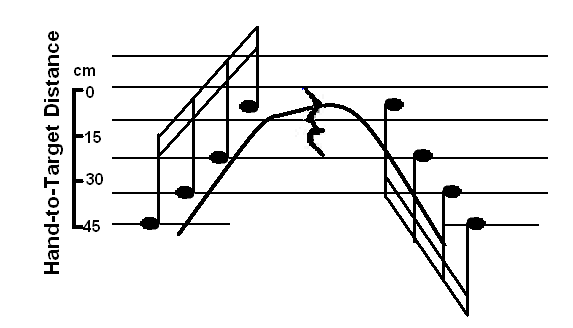
**Musical feedback design**. Musical notes are distributed along the hand's path from initial location to the target. Reaching a particular distance triggers the corresponding note to play. The curve indicates the hand-to-target distance during the arm reaching and withdrawal [72].

A research team at ASU is developing an immersive multimedia environment for biofeedback therapy (Fig. 2). The visual feedback presents the arm configuration and ideal trajectory of the hand from initial location to target. A cone shaped object indicates the spatial error of the endpoint. If the spatial error is large and the hand moves outside the boundary (spatial limits) of the guiding cone, the transparency of the cone becomes reduced, i.e., the cone is more visible. This produces the KP that tells the patient to correct the error. In addition to the visual feedback, auditory feedback in the form of musical notes indicates the smoothness and temporal-spatial parameters of the endpoint trajectory to improve multi-joint coordination (Figure 3) and to map the movement quality of the participant in real-time. Music notes within a phrase are distributed spatially along the specified path. These notes indicate the distance the hand has moved toward the target, with each note corresponding to a short distance along the path. When the hand reaches a point along the path where a musical note is located, the corresponding note starts to play. The duration of each note depends on movement speed. Therefore, patients could "compose" different melodies based on movement pattern and quality. If the movement is spastic, for example, the musical phrase could be distorted by multiple repetitions of a note. This music will play the same role as beeps in pacing the patient, but it will also provide information on speed and smoothness. In addition, another music phrase monitors trunk motion to signal the patient to reduce the compensatory motion in reaching [70]. In this case the volume indicates the amount of compensation.

Finally, haptic feedback has also been developed for task-oriented biofeedback studies [33,61,69,79,88-91]. Haptic interfaces allow the patient to interact with and to manipulate a virtual object. Results [69,89] have shown that haptic information provides knowledge of results (KR) and feeds back kinaesthetic sensations that are important for task performance. Haptics also encourage patients to immerse themselves in the virtual environment [61]. Haptic devices include the six degree of freedom (DOF) Cybergrasp from Immersion Corporation [89], the PHANTOM Haptic Interface [92], the 3DOF Haptic Master from Fokker Control Systems [79], and the Rutgers Master II-ND (RMII) force feedback [69,92].

Motivation and attention are two key factors in the success of therapies to induce neuroplasticity [93]. In earlier biofeedback approaches, the information presented often took the form of lines or bars on a computer screen or simple beeps. These were neither intuitive nor attention grabbing. Such feedback often makes participants, especially children, tire or become distracted quickly [25]. Novel VR based biofeedback systems can promote sustained attention, self-confidence, and motivation of participants during the repetitive task therapy through multimodal immersive displays and interactive training programs [79,90,94]. In some studies, the scenarios were also designed as games, such as goal keeping [94] or tennis playing [5], in an effort to engage the patient's active participation.

Finally, VR used as an integrated information technology can increase the patient's ability to process perceptual information in multisensing task-oriented biofeedback applications [95]. In virtual environments, the multimodal sensory cues that feed back multiple flows of information are presented to resemble scenes in the "real world" or in nature. Such an intuitive form of feedback is more easily perceived by brain injured patients than multiple abstract and quantified presentations that use formulas or numerics. Because the multisensing biofeedback system for functional retraining must also solve the information overloading problem, multimodal VR based feedback display offers a promising alternative approach.

There are other advantages of VR technology in task-oriented biofeedback as well. The virtual environment and training tasks are easily customized by the computer program [5]. Also, VR technology can assess the motor function recovery of patients [96,97]. Piron and co-workers [96] showed that objective measurements of task performance in VR produced high sensitivity and repeatability. Moreover, the augmented feedback, i.e., KR and KP, displayed in virtual environment can improve motor learning [50,98]. KR indicates the degree to which the performer achieved the desired movement outcome or therapeutic goal. KP is the augmented feedback of the quality of produced performance [50]. Virtual training environments can easily display both forms of feedback to inform patients about instant errors in task performance, motivate patients in task learning, and reinforce previous gains [50].

#### The application of Virtual Reality based task-oriented biofeedback

The major application of VR-based biofeedback to treat sensorimotor deficits has focused on UE exercise. Preliminary studies on VR based biofeedback for motor functional recovery in neurally injured patients are promising. Holden *et al*. [56] utilized VR to train reaching and hand orientation in stroke patients. Patients saw a virtual mailbox with different slot heights and orientations. To put the "mail" into the slot, the patient must reach the slot with correct hand orientation. A virtual "teacher mail" demonstrated the motion for patients to imitate. In preliminary findings, one of two stroke patients improved their upper extremity Fugl-Meyer Test (FM) score and their performance in a real mailing task. That patient was also able to complete some functional activities that previously were impossible. No improvement was observed in the other patient, however. Later, nine more participants were recruited for further testing [82]. All participants showed significant improvement in their FM score, the Wolf Motor Function score (WMF), and selected strength tests as compared to before the training. The study contained no comparison control or alternative treatment group, however. Also, the study provided no data or discussion on what parameters may affect the outcome. The inconsistent results in one patient [56] suggest that a single VR system design may not be effective for all stroke survivors. Wann and Turnbull [5] developed game-like VR based biofeedback programs to improve the amplitude and direction control of arm movement kinematics in eight adolescents with cerebral palsy. Each participant received two training sessions: VR based biofeedback and conventional occupational therapy, but in different orders. The researchers reported results from only three of the eight patients. In two patients with spastic diplegia, the VR-based biofeedback made the UE movement smoother than conventional therapy, as measured by the number of velocity peaks of elbow trajectory. No obvious benefit from VR-based training was observed in the third patient, who had severe athetosis. In studies investigating VR-based biofeedback for hand function rehabilitation in stroke survivors, investigators used multisensing data from the Cyberglove, which sensed finger joint angles, or the RMII glove, which measured both the applied force under each finger and the position of the fingertips [69,90]. Different scenarios were designed for exercises to improve joint range of motion, finger fractionation, and grasp strength on the impaired hand. Patients improved grasping force, finger joint range of motion, and movement speed after two weeks of VR-based biofeedback therapy [90]. Moreover, three participants showed an improvement on the Jebsen hand functional test [69]. This study focused on training of grasping movement and force, however, while the major impairment to hand function in stroke survivors is motor incapability for hand opening (extension of metacarpophalangeal joints) and wrist extension. In addition, pathological grasping, as seen in the tonic grasp reflex, for example, is common in brain injured patients. They may grasp the object tightly with finger flexion and adduction of thumb but then cannot release the object [99]. To improve the effectiveness of hand functional recovery in patients with brain injury, future designs of VR based biofeedback should emphasize motor tasks that encourage hand opening and wrist extension rather than retraining hand closure.

The number of reported VR-based biofeedback studies on LE motor function is relatively small at present, possibly due to the technical challenge of how to process a multitude of information and then present it to the patient. LE functional retraining depends not only on lower-limb mobility and bilateral coordination, but also requires other motor skills, such as balance control. A recent study used a video camera to track stroke patients' 2D motion and then fed back the motion by directly projecting and integrating the patient's image into a 2D game-like VR (IREX System, Toronto, ON, Canada) [100]. The experimental group included five stroke survivors. Each played three VR games with the goal of training LE range of motion, balance, mobility, stepping, and ambulation skills. KR and KP, such as error rate or movement quality, were quantified and indicated on the screen at the end of each game. The control group, also five stroke patients, did not receive any intervention. The experimental group significantly improved motor function in the LE and significantly increased activity in the primary sensorimotor cortex as determined by functional MRI data [100]. The investigators concluded that VR may have contributed to positive changes in neural reorganization and associated functional ambulation.

One inherent limitation of biofeedback therapy is that patients with more severe motor deficits cannot participate due to an inability to initiate any functional movement, thus preventing utilization of biofeedback for improving performance. Rehabilitation robots or other devices could solve the problem by providing mechanical assistance for movement.

Active research and development designs for robotic-focused UE or LE motor rehabilitation exist [101-105]. Several research groups have built robots with biofeedback features. One study used a robotic end-effector to help patients with stroke move their arms while receiving real-time feedback of endpoint position [79]. The robot could produce the force needed to correct the participant's hand position when it was out of the appropriate range. In gait training with Lokomat^®^, which can predefine the pattern of LE kinematics, estimation of self-generated joint torques is fed back. This provides information about the walking effort and motivates the patient to produce better gait patterns [106]. The combination of rehabilitation robot assisted therapy with advanced biofeedback is such an attractive approach for sensorimotor rehabilitation that we anticipate many new studies will forthcoming.

### Other techniques

Advanced technical developments in communication, including wireless vehicles and Internet use, have the potential to permit implementation of task-oriented biofeedback anytime and anywhere, thus enabling telerehabilitation. Jovanov et al. designed a wireless body area network that connects data from multiple sensors on the body to a personal server such as a cell phone or personal digital assistant (PDA) [66]. This data could be sent to other computers through a wireless network. The researchers suggested that this equipment could be used to provide biofeedback during ambulatory settings and to monitor trends during recovery. Another study presented an in-home biofeedback system in which many patients could access the same server via a telemedicine network. The VR based biofeedback training program could be customized to each participant, and retraining could then be performed using a personal computer at home [73]. Each provider would need many clients to establish a database and to keep records for each client efficiently while concurrently generating customizable training protocols to fit individual requirements [73]. The database would also be accessible to clinicians for evaluation of patient compliance and improvement. All these technological developments allow us to foresee the prevalence of task-oriented biofeedback applications in neurorehabilitation.

## Further considerations for task-oriented biofeedback

In the future, researchers should carefully choose the applied sensors and assign necessary biofeedback indexes in task-oriented biofeedback training. On one hand, the system should incorporate sufficient numbers and types of sensors to accurately detect the state of dynamic variables during movement. On the other hand, many catastrophically injured patients requiring physical therapy also have impaired perceptual and cognitive abilities. Therefore, it is important to develop an information fusion algorithm and to carefully design intuitive forms of feeding back integrated sensor information to avoid overloading patients' perceptions. In general, investigators should determine the factors contributing to motor deficits in each patient diagnostic group, establish training goals, explore the parameters that characterize functional movement, and then limit the number of feedback sources within the dynamic biofeedback paradigm.

The key ingredients for motor functional recovery are the intensity of task training and the patient's active involvement during the therapy [61]. Task-oriented biofeedback therapy and robot or other assistive device aided repetitive task practice should be more effective because this integrated sensorimotor therapy would provide patients with motor deficits an opportunity to actively and repetitively practice a task [79,107]. VR based displays could also increase the motivation and attention of patients in the task training, improve sensorimotor integration through multimodal augmented feedback, and, consequently, improve training efficiency. Therefore, comparing the effects of robot-aided therapy with task-oriented biofeedback intervention to conventional therapy for enhancement of motor function could be enlightening.

The application of virtual reality among patients with neuropathology is limited, however. VR-based biofeedback therapy requires that the patient demonstrate preservation of some auditory and/or cognitive ability or possess reasonable visual field perception. A certain level of movement control is also necessary to carry out tasks in the virtual environment. Immersive visual interfaces have been reported to increase the risk of seizure occurrence in patients with a history of epilepsy [108]. Pre-screening of participants should be performed based on clearly defined inclusion/exclusion criteria.

The most challenging question for all VR studies is whether the effect of VR training is transferable to task performance in the real world. If this transition cannot be acquired, VR may not be applicable in motor rehabilitation. Further evidence is needed to effectively address this question. Additionally, therapeutic goals from VR based studies need to be clinically and functionally relevant to be credible. For example, flexor spasticity develops in the hemiplegic hands and wrists of most patients with brain injuries [109]. Therefore, difficulties in opening the metacarpophalangeal and interphalangeal joints and extending the wrist are the relevant clinical problems, not the ability to close the hand for grasping, as one previous study attempted to correct [67,86]. Another problem with grasping task performance is that patients with brain injuries may have difficulty releasing objects due to uninhibited grasp reflexes [99]. Failure to release the grasped object during repetitive task training usually frustrates patients and may adversely impact motivation. Virtual reality offers an opportunity for patients to practice hand opening to release objects without haptic sensation, which avoids the tonic grasp reflex. Therefore, aVR based biofeedback system that trains patients to grasp and release virtual objects without haptic feedback may be effective to enhance the mobility of the hand, increase the active range of motion in metacarpophalangeal, interphalangeal and wrist joints, and motivate patients to practice hand opening activities in the early stages of the intervention. When patients regain active control of hand movement, haptic feedback could be added to the VR to enhance learning and interaction with the environment.

Preliminary results of clinical tests have demonstrated the benefits of task-oriented biofeedback on motor functional recovery [26,29,69,82,100]. However, these studies lack strong evidence. The number of patients in each study is small. Some reported benefits from task-oriented biofeedback were not consistently observed among all subjects [56]. Moreover, some of the applied technologies are immature. Clearly, future work should focus on techniques to enhance and ultimately foster RCTs directed toward task-oriented biofeedback applications. These RCTs should then use comprehensive statistical analyses to further prove and quantify the efficacy of task-oriented biofeedback for functional motor recovery.

## Conclusion

This article reviewed recent developments in biofeedback concepts, technologies, and applications. New technology propels the application of diverse biofeedback therapy options within the context of functional training to improve motor control among neurorehabilitation patients. Promising techniques for task-oriented biofeedback study, both developed and proposed, were summarized. Some preliminary clinical tests offer encouraging results. However, these techniques are relatively new, so there is a dearth of clinical RCTs available to definitively prove the efficacy of using contemporary technologies for task-oriented biofeedback within the field of neurorehabilitation. Further studies are needed.

## Abbreviations

UE: Upper ExtremityLE: Lower Extremity

IME: Interactive Multimodal environment

EMG: Electromyogram

CT: Conventional Therapy

ADL: Activities of Daily Living

RCT: Randomized Controlled Trial

VR: Virtual Reality

HMD: Head Mounted Display

DOF: Degree of Freedom

KP: Knowledge of Performance

KR: Knowledge of Result

FM: Fugl-Meyer Test

WMF: Wolf-Motor Functional Score

PDA: Personal Digital Assistant
